# Effect of Zinc on Spermatogenesis and Sperm Chromatin Condensation in Bleomycin, Etoposide, Cisplatin Treated Rats

**DOI:** 10.22074/cellj.2019.5522

**Published:** 2018-08-07

**Authors:** Shahnaz Razavi Razavi, Farnaz Khadivi, Fatemeh Hashemi, Abbas Bakhtiari

**Affiliations:** 1 Department of Anatomical Science, School of Medicine, Isfahan University of Medical Sciences, Isfahan, Iran; 2 Department of Anatomical Science, School of Medicine, Tehran University of Medical Sciences, Tehran, Iran

**Keywords:** Chemotherapy, Chromatin, Seminiferous Tubules, Spermatozoa, Zinc

## Abstract

**Objective:**

The incidence rate of testicular cancer among young males is high. Co-administration of bleomycin, etoposide and cisplatin (BEP) has increased survival rate of patients with testicular cancer. Although BEP is one of the most effective treatment for testicular cancer, but it severely affects the reproductive system that ultimately leads to infertility. In addition to its antioxidant activity, zinc has an important role in progression of spermiogenesis. This study aimed to evaluate the effect of zinc on sperm parameters, chromatin condensation and testicular structure after BEP treatment.

**Materials and Methods:**

In this experimental study, 40 male rats were divided into 4 groups (control, BEP, BEP+ zinc and zinc) and examined for 2 spermatogenesis periods (i.e. 18 weeks). The rats in BEP and BEP+ zinc group were treated with BEP at appropriate doses (0.75, 7.5, and 1.5 mg/kg) for three cycles of three weeks. Zinc at a dose of 10 mg/kg/day was administered to BEP+ zinc and zinc groups. After 18 weeks, we assessed sperm parameters, and excessive histone in sperm chromatin using aniline blue staining, as well as testicular structure and germ line cells using periodic acid-Schiff staining.

**Results:**

After BEP treatment, significant decreases were observed in normal sperm morphology, motility, and concentration, as well as alterations in rat sperm chromatin condensation and testicular tissue (P<0.001). Furthermore, after zinc consumption for 9 weeks, we observed significant improvements of sperm parameters and chromatin condensation as well as a significant retrieval of spermatogonia, leydig cells and tubular architecture (P<0.05).

**Conclusion:**

Zinc administration after chemotherapy with BEP in testicular cancer might be potentially useful in declining the off target consequence associated with oxidative stress.

## Introduction

Approximately 50% of infertility cases among couples are caused by male factors and have been shown to be associated with varicocele, cryptorchidism, infection, obstructive lesions in the genital tract, torsion and testicular tumors (1). As cited in previous studies, there is a direct correlation between changes in sperm parameters and male infertility; So, semen analysis is an important tool for evaluation of infertility in men (2). 

Oxidative stress is an effective factor that has been widely studied as it can influence fertility potential (3). Presence of reactive oxygen species (ROS) at physiological levels is essential for sperm functions such as acrosomal reaction and capacitation (4). It is also necessary for reproductive processes such as the interaction between sperm and oocyte (5) and the implantation and initial development of the fetus (6).

Research has shown that infertile people have low antioxidant potential and high levels of ROS in their semen(7). Due to the high metabolism and cell proliferation intesticular tissue, oxidative stress can severely damage thisorgan, in spite of the fact that spermatogenic cells are protectedby the blood-testis barrier (8). In addition, it has been shownthat oxidative stress can damage the basement membrane of the seminiferous tubules and affect spermatogenesis (9). 

Chemotherapy is one of the factors that induces infertility (10), and chemotherapeutic drugs through induction of oxidative stress, lipid peroxidation and cell apoptosis cause cell death (11).

BEP chemotherapy (bleomycin, etoposide and cisplatin) is one of the standard treatments for advanced testicular cancer (10). Bleomycin binds to DNAgaps in the presence of iron and oxygen molecules (11), through the following mechanisms: i. Detecting a specific base or base sequence on DNA, ii. Generation of ROS, and iii. Induction of oxidation reactions causing DNA breakage (12). Etoposide targets topoisomerase II, an enzyme that changes the topology of DNA(13). Cisplatin is an alkylating agent which binds an alkyl group to the DNA molecule and prevents DNA replication (14).

According to previous studies, BEP chemotherapeutic agents, in addition to destroying cancerous cells through ROS production, lead to DNA damage, DNA breaks, disruption of seminiferous tubules and testicular structure, and changes in levels of blood hormones (15, 16). BEP treatment leads to disturbances in the displacement ofhistone with protamine, which impair spermiogenesis and chromatin condensation (17). 

Zinc affects essential physiological processes such as cellular response to oxidative stress, DNA repair, cell cycleand apoptosis (18). It is an essential part of the superoxidedismutase enzyme, which converts superoxide anion into H_2_O_2_ and oxygen molecules, it is therefore an important antioxidant (19).

Since replacement of histone with protamine is one of theimportant protective strategies of sperm, we investigatedexcessive histone in sperm. In addition, we evaluated degenerative alterations in testicular tissue after treatmentwith BEP, and assessed compensatory effects of zinc on the aforementioned factors, both after chemotherapy and alone. 

## Materials and Methods

### Experimental animals

In this experimental study, 40 mature male Wistar rats (180250 g and 14 weeks old) were procured from Pasteur Institute, Tehran, Iran and used in this study. All animal experimentswere approved by Isfahan University of Medical Sciences’Ethics Committee. All rats were kept at 18-24°C, with 12hours light/12 hours dark cycles. Rats had free access to astandard rat chow and tap water ad libitum. Acclimatizationof animals was done for 10 days before initiation of the experiment. 

### Bleomycin, etoposide, cisplatin and antioxidant treatment procedure

Male rats were randomly divided into 4 groups of control, BEP treatment, BEP treatment then zinc and zinc with 10 rats in each group. The control (Sham) animals received an intraperitoneal injection of 1 ml of 0.9% saline for 18 weeks on days 1-5 of each week. The rats in BEP group initially received an intraperitoneal injection of 1 ml of 0.9% saline for 9 weeks and then received 7.5 mg/kg etoposide, 1.5 mg/ kg cisplatin on days 1-5 of each week. On day 2 of each week, rats received 0.75 mg/kg bleomycin for 9 weeks. Injections administered on the same day were performed with an interval of 30 minutes and all of drugs were dissolved in 0.9% saline (20). The rats in BEP and zinc group initially received BEP (similar to the BEP group) for 9 weeks and then zinc was administered as zinc sulfate heptahydrate similar to zinc group for 9 weeks (for a total of 18 weeks). The rats in zinc group were gavaged with 0.9% saline for 9 weeks and then administered with an oral gavage of 10 mg/kg/day zinc (diluted in saline) for 9 weeks (21). All the materials were purchased from Sigma-Aldrich, USA unless otherwise mentioned.

### Sperm collection

At the end of the experiment, the rats of four groups were anesthetized by intraperitoneal administrations of 90 mg/kg ketamine and 10 mg/kg xylazine. All rats were sacrificed under anesthesia. Epididymis were removed, and then adipose tissues were removed. The caudal epididymis was separated and placed in 1 mL of warm normal saline (37°C); then, several incisions were made on the epididymis and sperm suspension was placed in an incubator for 10 minutes

### Sperm parameters

The sperm concentration and motility were determined by phase-contrast microscope (Nikon TE2000-U, Japan) at magnification ×200. 

At first, 100 µl of semen suspension was added to 900 µl normal saline. An aliquot of the diluted suspension was put into the Neubauer chambers and head of sperm was manually counted. For sperm motility, data was expressed as percentage of motile and non-motile sperm. Sperm morphological abnormalities were evaluated by images obtained from phase-contrast microscope. For each sample, 200 sperm were evaluated for head, neck, and tail abnormalities (22, 23).

### Aniline blue staining

In order to determine sperm chromatin condensation, and assessment of excessive histone, aniline blue staining was performed. First, sperm smears were fixed in 3% buffered glutaraldehyde in 0.2 M phosphate buffer (14 ml Na_2_HPO_4_ 0.2 M+36 ml Na_2_HPO_4_ 0.2 M, pH=7.2) for 30 minutes. Next, the smears were stained with 5% aqueous aniline blue (Merck, Schuchardt, Germany) in 4% acetic acid (pH=3.5) for 5 minutes (24). Smears were assessed by light microscopy (Olympus CH30, Japan); dark blue sperm had excessive histone and considered positive while, light blue sperm had normal chromatin structure and considered negative. 

### Periodic Acid Schiff staining 

Left testis was fixed in a Bouin’s solution for 24 hours. Then, tissue processing was performed in an automatic processing machine. Sections of 5 µm were prepared, deparaffinized and then dehydrated. Sections were oxidized in 0.5% periodic acid solution for 5 minutes and washed in distilled water. Then, the sections were placed in Schiff’s reagent for 15 minutes and washed in tap water for 5 minutes. Finally, they were counterstained with Mayer’s haematoxylin for 1 minute, washed in tap water, dehydrated, cleared in xylol and mounted (25). Tissue analysis was done using light microscopy (Olympus CH30, Japan) and finally, the number of Leydig cells, spermatogonia and thickness of basement membrane were measured by imageJ software (Version1.240).

### Statistical analyses

The SPSS software version 22.0 was applied for data analyzing. All data are expressed as mean ± standard error of the mean (mean ± SE); Differences among means were considered statistically significant when P<0.05. Kolmogorov-Smirnov test was used to determine normal distribution of data, for multiple comparisons of data, One- Way ANOVA was applied.

## Results

According to Table 1, the mean percentage of sperm concentration in BEP group significantly decreased as compared to other groups. However, in BEP+ zinc group sperm concentration improved but did not reach that of the control group. In addition, the mean percentage of sperm concentration in zinc group was not significantly different from that of the control group.

**Table 1:** Comparison the mean percentages of sperm parameters in different experimental groups (control, BEP, BEP+ zinc and zinc)


Group	Control	BEP	BEP+ zinc	Zinc
Sperm parameters	Mean ± SE	Mean ± SE	Mean ± SE	Mean ± SE

Concentration (×10^6^)	58.50 ± 1.88^b^	33.03 ± 1.25	50.35 ± 1.41^a, b^	60.90 ± 1.76^b^
Motility (%)	73.73 ± 1.89^b^	36.48 ± 1.63	65.06 ± 2.33^a, b^	75.53 ± 2.11^b^
Abnormal morphology (%)	21.93 ± 1.33^b^	37.07 ± 1.48	28.26 ± 1.49^a, b^	20.40 ± 1.53^b^


^a^; Significant differences as compared to control group (P<0.05) and ^b^; Significant differences as compared to BEP group (P<0.001).

The mean percentage of sperm motility was significantly decreased in BEP group compared to control group (P<0.001). The mean percentage of motile sperm in BEP+ zinc significantly increased compared to BEP group (P<0.05); however, zinc group did not show significant differences compared to the control group. 

The mean percentage of total abnormal morphology in BEP group was increased compared to control group (P<0.001). Moreover, in BEP+ zinc group abnormal morphology of spermatozoa was recovered but it was still significantly different compared to that of control group (P<0.05). Whereas, the mean percentage of total abnormal morphology in zinc group was not significantly different from that of the control group.

**Fig.1:**
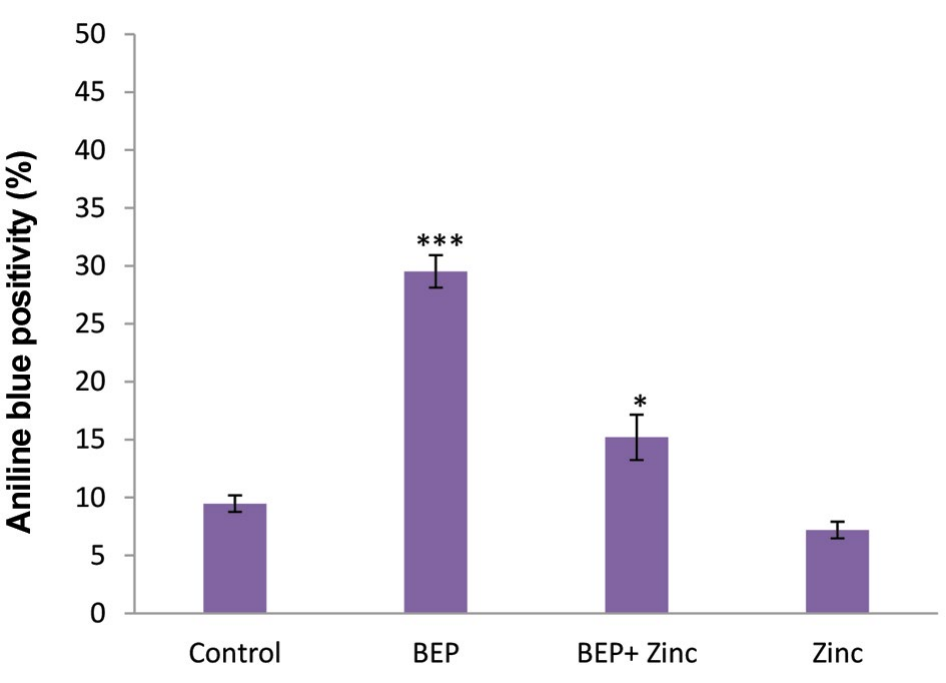
Comparative analysis the mean percentages of sperm with excessive histone (aniline blue positive sperm) in different groups.***; P<0.001 and *; P<0.05 show significant differences as compared to control group.

**Fig.2:**
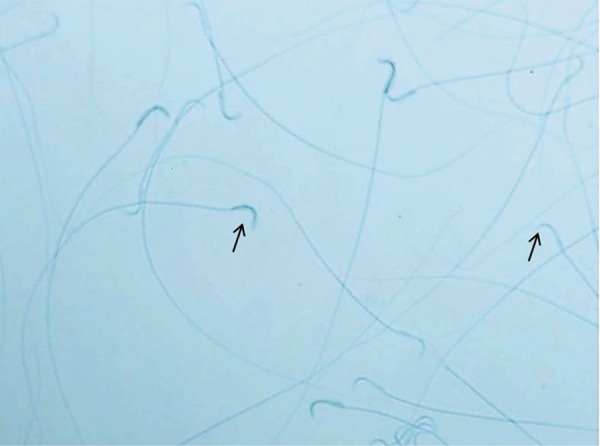
Photograph of aniline blue staining; sperm with excessive histone were dark blue stained (Left arrow), while sperm with correct histone-protamine replacement were light blue stained (right arrow).

In BEP group, the mean percentage of sperm with excessive histone (aniline blue positive) was significantly increased as compared to other groups (P<0.001, Fig .1). In BEP+ zinc group, excessive histone levels decreased but there was still significant differences compared to control group (P<0.05). However, zinc group had no significant differences compared to the control group in mean percentage of aniline blue positive sperm (Fig .2). 

**Fig.3:**
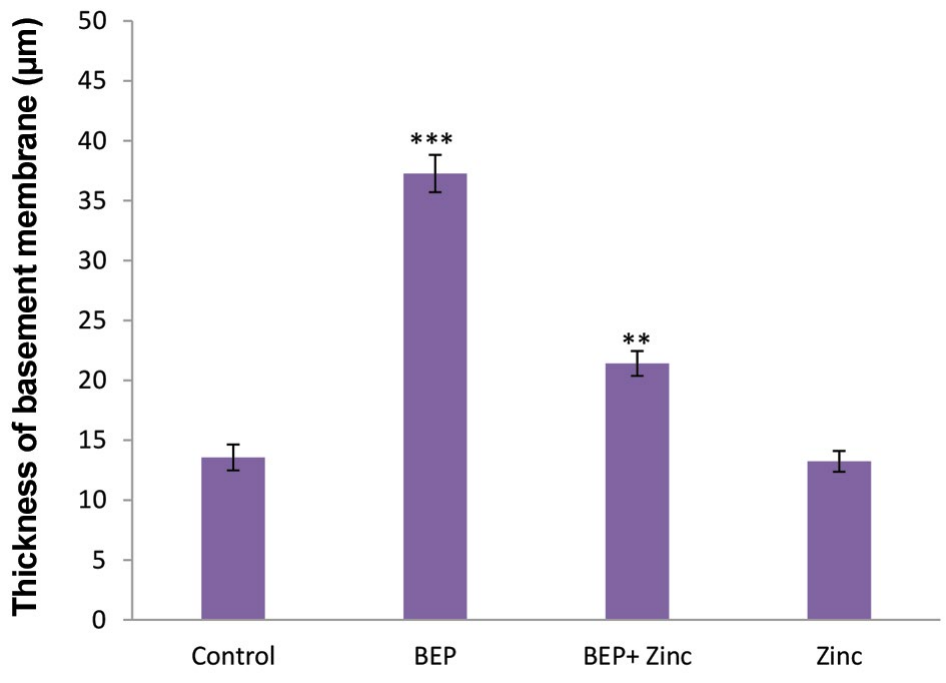
Comparative analysis of the mean thickness of seminiferous tubules basement membrane in rats of different groups. ***; P<0.001 and **; P<0.01 show significant differences as compared to control group.

**Fig.4:**
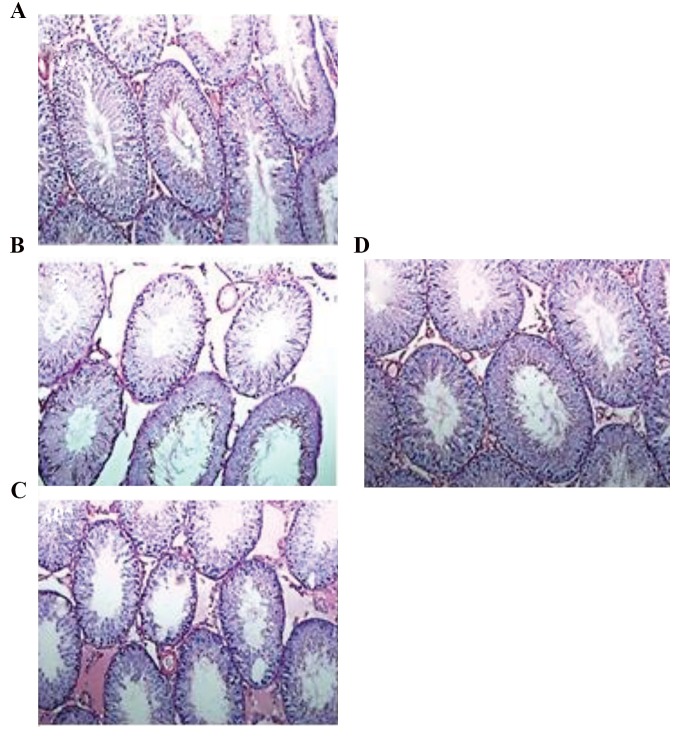
Photograph of rats testicular tissue stained with Periodic AcidSchiff (PAS), **A.** Control, **B.** BEP, **C.** BEP+ zinc, and D. Zinc group (scale bars: 200 µm).

One of the important factors examined in seminiferous tubules studies, is the normal structure of the basement membrane, the mean thickness of basement membrane in BEP group increased significantly compared to other groups (P<0.001), while in BEP+ zinc group the mean thickness of basement membrane decreased compared to BEP group, but not as much as the control group. However, there was still significant difference as compared to control group (P<0.01). But, the mean thickness of basement membrane in zinc group was not significantly different from that of the control group (Fig .3). After BEP treatment, detachment of basement membrane, disorganization and atrophy as well as vacuolization in some of the tubules were observed (Fig .4). 

**Fig.5:**
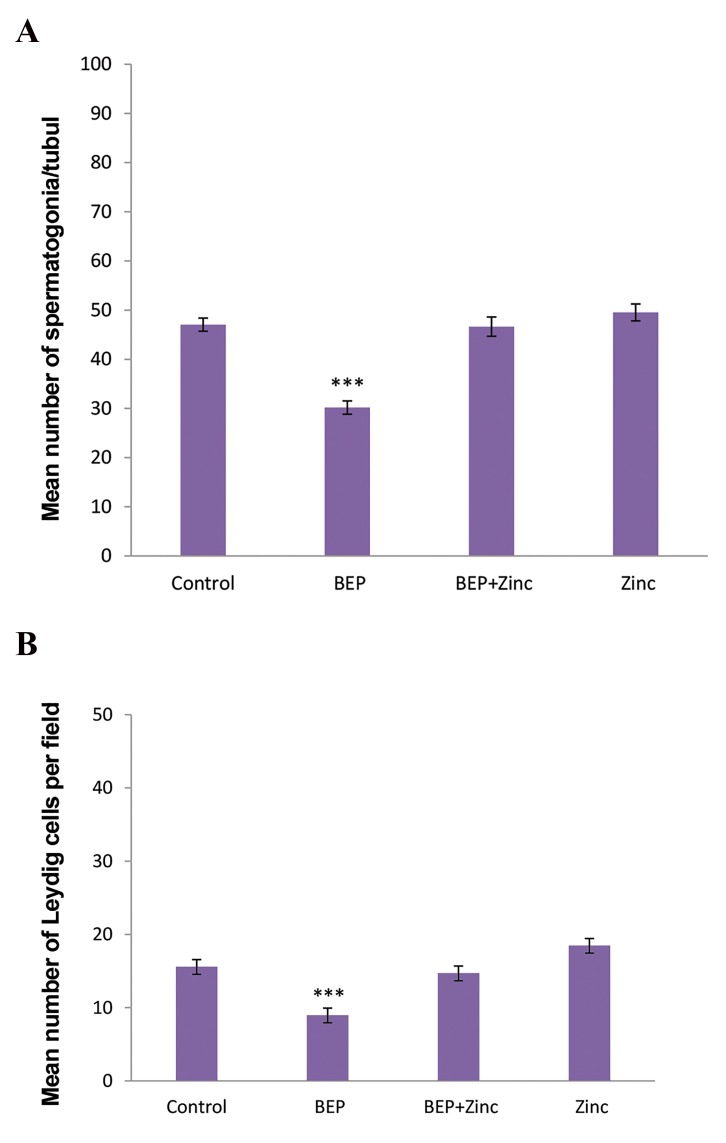
Comparison of the mean number of cells. **A.** Comparison of the mean number of spermatogonia per seminiferous tubule and **B.** Leydig cells per field in different groups. ***; P<0.001 shows significant differences as compared to control group.

By investigating PAS-stained sections, we observed that the mean numbers of spermatogonia and Leydig cells in BEP group were significantly lower than those of other groups (P<0.001). Although testicular germ cells and testicular tissue after zinc administration were recovered relative to BEP group, but the mean number of spermatogonia and Leydig cells in BEP+ zinc and zinc groups showed no significant differences compared to the control group (Fig .5).

## Discussion

Testicular cancer is the most common cancer that affects men of reproductive ages (26). One of the effective treatments for testicular cancer is BEPwhich includes three different drugs with three different mechanisms of action on cancerous cells (27). Noteworthy, chemotherapeutic drugs are one of the known causes of infertility among people.

In this study, for the first time, we evaluated the effect of zinc, as an antioxidant with a wide range of beneficial properties on the sperm parameters, chromatin condensation and histological structure of the testis. Also, in this study, we used a cancer-free animal model in order to prevent any confounding that may be induced by cancerous germ cells in the testis. There is a controversy concerning the simultaneous use of antioxidants with chemotherapeutic drugs. Since concurrent use of antioxidants and chemotherapeutics can interfere with the anti-cancer performance of drugs, unlike other previous studies, we used zinc after the chemotherapy period (11, 28, 29)

In the present study, the duration of chemotherapy and duration of zinc intake after chemotherapy considered as three cycles of three weeks, because one spermatogenesis period in the rat completed and the effects of the drug and zinc can be checked properly. Normal sperm parameters are important factors in primary assessment of male fertility potential and are evaluated through semen analysis. In BEP+ zinc group, the mean percentages of sperm motility, concentration and morphology were improved compared to BEP group, but they were significantly different from those of the control group. Probably, for this reason, patients were advised to avoid conception during the treatment for 2 years after the end of therapy in order to prevent the transmission of the defective genome to the next generation (30, 31). 

The results of aniline blue staining showed a significant increase in the presence of sperm with excessive histone after chemotherapy which coincided with previous findings from chromomycinA3 (CMA3) and toluidine blue staining (21). Zinc administration after chemotherapy induced a significant recovery in terms of replacement of histone by protamine, since the presence of zinc is necessary for the formation of bi-sulfide bonds in the process of spermiogenesis. Therefore, zinc intake, not only as an antioxidant, also due to its importance in progression of spermiogenesis, leads to improvement of chromatin integrity. Correct replacement of protamine for histone and consequently, condensation of sperm chromatin is necessary for protection of the sperm chromatin against damages (15).

In previous studies, the effects of chemotherapeutic agents and antioxidants on the histological structure of the testicles and germ cell line, were investigated (20, 29). In this study, for the first time, we examined effect of chemotherapeutic agents and zinc on mean thickness of the basement membrane in testicular sections following PAS staining. After BEP treatment, disarrangement in seminiferous tubules and a significant decrease in germ line cells, were observed. 

Unlike other germ cells, in BEP group, we observed nucleus of spermatogonia that indicated higher resistance of these cells towards environmental stress and their survival confirmed recovery of fertility after the end of treatment. Partial recovery after zinc consumption in BEP+ zinc group indicated compensatory effects of zinc beside 9 weeks recovery after BEP treatment. Although the number of germ cells in BEP+ zinc group significantly increased compared to BEP group, more vacuolization in the epithelium of the seminiferous tubules and detachment of basement membrane in the BEP+ zinc group can be regarded as a long-term complication of chemotherapy drugs.

## Conclusion

According to our study, zinc efficiently ameliorates the consequences of BEP treatment in terms of sperm parameters, proper chromatin condensation and testicular structure. These outcomes maybe due to role of zinc in spermiogenesis as well as its antioxidant effect. Therefore, zinc consumption after chemotherapy in patients with testicular cancer is suggested. 

## References

[1] Turner TT, Lysiak JJ (2008). Oxidative stress: a common factor in testicular dysfunction. J Androl.

[2] Kovac JR, Smith RP, Cajipe M, Lamb DJ, Lipshultz LI (2017). Men with a complete absence of normal sperm morphology exhibit high rates of success without assisted reproduction. Asian J Androl.

[3] Geva E, Lessing JB, Lerner-Geva L, Amit A (1998). Free radicals, antioxidants and human spermatozoa: clinical implications. Hum Reprod.

[4] Valko M, Morris H, Cronin MT (2005). Metals, toxicity and oxidative stress. Curr Med Chem.

[5] De Lamirande E, Leclerc P, Gagnon C (1997). Capacitation as a regulatory event that primes spermatozoa for the acrosome reaction and fertilization. Mol Hum Repro.

[6] Sakkas D, Urner F, Bizzaro D, Manicardi G, Bianchi P, Shoukir Y (1998). Sperm nuclear DNA damage and altered chromatin structure: effect on fertilization and embryo development. Hum Reprod.

[7] Zini A, de Lamirande E, Gagnon C (1993). Reactive oxygen species in semen of infertile patients: levels of superoxide dismutase‐and catalase‐like activities in seminal plasma and spermatozoa. Int J Androl.

[8] Richburg JH, Boekelheide K (1996). Mono-(2-ethylhexyl) phthalate rapidly alters both Sertoli cell vimentin filaments and germ cell apoptosis in young rat testes. Toxicol Appl Pharmacol.

[9] Yang HS, Han DK, Kim JR, Sim JC (2006). Effects of alpha-tocopherol on cadmium-induced toxicity in rat testis and spermatogenesis. J Korean Med Sci.

[10] Zanchi MM, Manfredini V, Brum DDS, Vargas LM, Spiazzi CC, Soares MB (2015). Green tea infusion improves cyclophosphamideinduced damage on male mice reproductive system. Toxicol Rep.

[11] Kopp HG, Kuczyk M, Classen J, Stenzl A, Kanz L, Mayer F (2006). Advances in the treatment of testicular cancer. Drugs.

[12] Cort A, Ozben T, Melchiorre M, Chatgilialoglu C, Ferreri C, Sansone A (2016). Effects of bleomycin and antioxidants on the fatty acid profile of testicular cancer cell membranes. Biochim Biophys Acta.

[13] Ehrenfeld GM, Rodriguez LO, Hecht SM, Chang C, Basus VJ, Oppenheimer NJ (1985). Copper (I)-bleomycin: structurally unique complex that mediates oxidative DNA strand scission. Biochemistry.

[14] Nitiss JL (2009). Targeting DNA topoisomerase II in cancer chemotherapy. Nat Rev Cancer.

[15] Wang D, Lippard SJ (2005). Cellular processing of platinum anticancer drugs. Nat Rev Drug Discov.

[16] Maselli J, Hales BF, Robaire B (2013). The effects of chemotherapy with bleomycin, etoposide, and cis-platinum (BEP) on rat sperm chromatin remodeling, fecundity and testicular gene expression in the progeny. Biol Reprod.

[17] Ghezzi M, Berretta M, Bottacin A, Palego P, Sartini B, Cosci I (2016). Impact of Bep or carboplatin chemotherapy on testicular function and sperm nucleus of subjects with testicular germ cell tumor. Front Pharmacol.

[18] Bagheri-Sereshki N, Hales BF, Robaire B (2016). The effects of chemotherapeutic agents, bleomycin, etoposide, and cisplatin, on chromatin remodeling in male rat germ cells. Biol Reprod.

[19] Ho E (2004). Zinc deficiency, DNA damage and cancer risk. J Nutr Biochem.

[20] Narayana K (2008). An aminoglycoside antibiotic gentamycin induces oxidative stress, reduces antioxidant reserve and impairs spermatogenesis in rats. J Toxicol Sci.

[21] Kilarkaje N, Mousa AM, Al-Bader MM, Khan KM (2013). Antioxidants enhance the recovery of three cycles of bleomycin, etoposide, and cisplatin-induced testicular dysfunction, pituitary-testicular axis, and fertility in rats. Fertil Steril.

[22] Maremanda KP, Khan S, Jena G (2014). Zinc protects cyclophosphamideinduced testicular damage in rat: Involvement of metallothionein, tesmin and Nrf2. Biochem Biophys Res Commun.

[23] Narayana K, Verghese S, Jacob SS (2009). L-Ascorbic acid partially protects two cycles of cisplatin chemotherapy-induced testis damage and oligo-astheno-teratospermia in a mouse model. Exp Toxicol Pathol.

[24] Narayana K, Al-Bader M, Mousa A, Khan KM (2012). Molecular effects of chemotherapeutic drugs and their modulation by antioxidants in the testis. Eur J Pharmacol.

[25] Razavi S, Nasr‐Esfahani MH, Mardani M, Mafi A, Moghdam A (2003). Effect of human sperm chromatin anomalies on fertilization outcome post‐ICSI. Andrologia.

[26] Celik-Ozenci C, Bayram Z, Akkoyunlu G, Korgun ET, Erdogru T, Seval Y (2006). Localization of NGF and nNOS in varicocele-induced rat testis. Acta Histochem.

[27] Schmoll HJ, Jordan K, Huddart R, Pes MP, Horwich A, Fizazi K (2010). Testicular seminoma: ESMO Clinical Practice Guidelines for diagnosis, treatment and follow-up. Ann Oncol.

[28] Einhorn LH, Foster RS (2006). Bleomycin, etoposide, and cisplatin for three cycles compared with etoposide and cisplatin for four cycles in good-risk germ cell tumors: is there a preferred regimen?. J Clin Oncol.

[29] Uygur R, Aktas C, Tulubas F, Uygur E, Kanter M, Erboga M (2014). Protective effects of fish omega‐3 fatty acids on doxorubicin‐induced testicular apoptosis and oxidative damage in rats. Andrologia.

[30] Tempest HG, Ko E, Chan P, Robaire B, Rademaker A, Martin RH (2007). Sperm aneuploidy frequencies analysed before and after chemotherapy in testicular cancer and Hodgkin’s lymphoma patients. Hum Reprod.

[31] Ping P, Gu BH, Li P, Huang YR, Li Z (2014). Fertility outcome of patients with testicular tumor: before and after treatment. Asian J Androl.

